# Environmental regulation, environmental responsibility, and green technology innovation: Empirical research from China

**DOI:** 10.1371/journal.pone.0257670

**Published:** 2021-09-22

**Authors:** Yuanyang Wang, Yanlin Yang, Chenyu Fu, Zengzeng Fan, Xiaoping Zhou

**Affiliations:** 1 Economics and Management School, Wuhan University, Wuhan, Hubei, China; 2 Center for Economic Development Research and Center of Population, Resource & Environmental Economics Research, Wuhan University, Wuhan, Hubei, China; 3 Institute for Advanced Studies in Finance and Economics, Hubei University of Economics, Wuhan, Hubei, China; 4 Business School, Zhengzhou University, Zhengzhou, Henan, China; Institute for Advanced Sustainability Studies, GERMANY

## Abstract

**I**nnovation and green are the directions to promote the circular economy and environmental sustainability at the corporate level. This paper examines the impact of environmental regulation (pollution charge) on green technology innovation and the mediating role of corporate environmental responsibility. Our results indicate that: (1) Environmental regulations stimulate manufacturing enterprises’ environmental responsibility and green technology innovation. It is worth noting that corporate environmental responsibility strengthens the relationship between environmental regulation and green technology innovation. (2) Further investigation reveals that R&D expenditure and environmental investment have greatly strengthened the positive effect of environmental regulation on green technology innovation. (3) With more detailed disclosure about enterprises’ environment-related information, the more outstanding stimulation effects of environmental regulation. Discussions on the features of enterprise location have revealed that, if the goal of environmental protection is set too high or if the fiscal decentralization is too strong, implementation of environmental regulation would not achieve desirable results. Accordingly, we need to optimize the collection of environmental taxes, strengthen the enterprises’ environmental responsibility, and increase investment in R&D and environment protection. Meanwhile, the execution of environmental regulation should also take into account the institutional environment and governance features of the enterprise locations.

## Introduction

The key projects faced in contemporary China are to accelerate the green transformation of development modes and to achieve high-quality economic development. The green innovation capabilities and environmental responsibility levels of heavily polluting listed companies in China are relatively low. Therefore, the government has adopted various means of environmental regulations to restrain the enterprises, to form a green, efficient, and economical way of production. Then, did environmental regulation has improved corporate environmental responsibility and green innovation activities? Corporate environmental responsibility (CER) means that enterprises take the responsibility that benefits sustainable development. Green innovation runs through the entire process of an enterprise’s production, consumption, and service, involving renovations of technology, organization, and institutions [1]. Existing studies on environmental regulation are mostly conducted on the level of regions and industry, lacking in-depth discussion on the environmental regulation effects and inner mechanism of enterprises [2]. It is of great significance to evaluate the response of manufacturing enterprises to environmental regulation policies. We examine the impact of environmental regulation on green technology innovation (GTI) and the mediating effect of CER. This paper also presents the heterogeneous effect of environmental regulations on innovation in different governance environments, thereby providing a theoretical basis for promoting the green development of manufacturing enterprises.

Numerous scholars have agreed that the economic effects of environmental regulations from two major perspectives, namely the “compliance cost” and the “Porter Hypothesis” [2–5]. On the one hand, pollution control action has consumed large amounts of resources for manufacturing enterprises. Enterprises may have to cut down on R&D spending to improve their financial situation, and innovation may not be the primary option for them to implement green development [3,4]. On the other hand, Porter [5] pointed out that moderate environmental regulation stimulates corporate innovation and creates an innovation compensation effect. In particular, market-incentive environmental policies like pollution charges and carbon permits trading will enhance an enterprise’s competitiveness by inspiring technology innovation [6,7]. Kesidou and Wu [8] suggest that stricter environmental regulatory frameworks in emerging economies are not only combating pollution but also shifting the innovation activities of manufacturing firms. For heavily polluting companies, a proactive environmental strategy and energy policy will help promote enterprises’ energy-saving transformation and improve their future performance [9,10]. Acemoglu et al. [11] believe that the cooperation of two environmental regulation tools—pollution emission charges and environment subsidies—can achieve enterprises’ green technology innovation. With the spread and reinforcement of environmental regulations, green technology innovation holds a better advantage for enterprises [12].

Firms’ correct choices on environmental governance methods and implementation strategies can arguably have a positive impact on their innovation capacity [13,14]. Various studies establish that the interlinkages of CSR, CER, and innovation, studying the impact of CSR and CER on the economic consequences of enterprises [15–18]. From the perspective of stakeholder theory, stakeholders have a markedly impact on the industry’s greening [19]. CSR can help firms to gain external resources, such as government support and social support [16]. Imed et al. [20] hold that corporate innovation is driven only by the environmental dimension of CSR. As the main part of CSR, CER is the result of enterprises responding to the government’s environmental regulations [21]. CER depends on environmental management strategies, and a better effective environmental governance is beneficial for enterprise performance [22–24]. Ferri [25] found that enterprises with a stronger social responsibility are more likely to make green investments. Liu et al. [26] believe that improvement of governance structure may enhance heavily polluting firms’ environmental performance and eventually promote their capability of sustainable development. While inefficient corporate governance may bring down the enterprise’s environmental performance and inhibit its green innovation [27]. In this sense, firms focusing on CER can capture stakeholders’ preferences, turning external resources into innovation support.

Environmental regulations affect enterprises’ green technology innovation via more than one route. However, there has not been much research on the internal mechanism of environmental regulations affecting corporate behavior, especially from the perspective of environmental responsibility. Enterprises can choose non-green innovation, green innovation, and take environmental responsibility when subject to environmental regulation. Heavily polluting manufacturing enterprises have to enhance their inner governance to improve environmental performance. In particular, environmental regulation stimulates environmental management certification and improves environmental responsibility [28]. As an important part of social responsibility, environmental responsibility is becoming a decisive factor influencing company green performance.

This paper focuses on the impact of environmental regulation on manufacturing enterprises’ green technology innovation, analyzing the regulating functions of R&D expenditure and environmental protection investment. We find that environmental regulation has a positive effect on corporate green technological innovation. Part of the role of environmental regulation in promoting green innovation is achieved by strengthening corporate environmental responsibility. The conclusion still stands after robustness tests of replacing independent variables and econometric models. Further investigation reveals that the input of R&D and environment protection have both greatly strengthened the positive effect of environmental regulation on green technology innovation. The environmental information disclosure will enhance the positive effect of pollution charges on green technological innovation. In addition, excessive environmental protection goals or fiscal decentralization in the area where the company is located is not conducive to the implementation of environmental policies.

This study offers a two-fold contribution. First, this paper reveals the relationship between environmental regulation, corporate environmental responsibility, and green technology innovation. To this end, we shed light on the mediating effect of CER. Second, this paper explores the regulating effect of factors such as R&D expenditure and environment protection investment, thus offering theoretical guidance for enterprises to deal with environmental regulation. By studying the regional differences concerning environmental regulation’s impact on green technology innovation, this paper puts forward policy recommendations for coordinated growth of regional economy and environment.

The remaining parts of this paper are organized as follows: Section 2 provides the formulation of the research hypotheses; Section 3 describes the quantitative analysis concerning (a) the sample selection and data sources, (b) the choice of the variables, (c) the econometric models; Section 4 sets out the empirical research findings and robustness test; Section 5 provides further research and heterogeneity analysis; Section 6 provides the discussion of findings and conclusions.

## Theoretical background and hypotheses development

### Environmental regulation and green technology innovation

Under the pressure of regional environmental regulations, enterprises have to pay pollution charge and increase investment in environmental governance. These environmental governance behaviors take up a lot of resources and create new production costs, which is not conducive to innovation input. In addition, strict environmental regulation measures will inhibit the financial activities of heavily polluting companies [29]. While some scholars believe that only the long-term benefits of innovation can compensate for the costs of environmental regulations. According to the Porter Hypothesis, moderate environmental regulation stimulates technology innovation and thereby bringing “innovation compensation” of increased competitiveness. The competitive advantage formed through technological innovation gains long-term profits for enterprises and maintains their market competitiveness. External knowledge adoption and green absorptive capacity strengthen the positive impact of environmental regulation [30]. Accordingly, the enterprise’s continuing innovation depends on the interaction between the “costs compliance effect” and the “innovation compensation effect”. Alongside the accelerated construction of ecological civilization, environmental regulation has become a standardized restraint on manufacturing enterprises, making innovation the only way for companies to gain a competitive advantage [31].

Unlike innovations of non-green technology, green innovation means the implementation of green concepts to the entire process of corporate production and operation. It involves innovation of green technology, green management, and green marketing. Green innovation promotes the sustainable development of enterprises by improving the technical level of products and reducing environmental pollution [32]. Heavily polluting manufacturing enterprises have to increase R&D investment for improving green innovation. Green innovation fundamentally enhances product competitiveness and brings competitive advantages to enterprises. Firstly, enterprises can obtain government’s environmental protection and innovation subsidies with technological innovation, which in turn brings a product’s marketing profits. Secondly, the pressure from stakeholders compels listed enterprises to implement green development strategies and green innovation into business management decisions. It can be inferred that environmental regulation will stimulate the enterprise to transform external pressure into a “motivating factor” encouraging innovation. Innovation is the starting point for a company to obtain the “compensation effect” of environmental regulation. For heavily polluting manufacturing enterprises, green technology innovation has far more competitive strengths compared with other forms of innovation. Ultimately it is the environmental regulation policy that encourages manufacturing enterprises to perform green technology innovation. Based on the above, this paper proposes the following hypotheses:

Hypothesis 1: Environmental regulation is positively associated with the green technology innovation of manufacturing enterprises.

### The mediating roles of corporate environmental responsibility

Companies will pay more attention to environmental responsibility under the influence of environmental regulation. CER including environmental protection investment, environmental information disclosure, green marketing, and these factors are conducive to innovation. Apart from direct supervision, the government will urge enterprises frequently to be more responsible via compulsory environment reports. Companies with better environmental performance will actively disclose environmental information in their social responsibility reports. [33]. Environment responsibility is a major component of corporate social responsibilities and is under the scrutiny of both stockholders and the capital market. As required by the *Company Law of the People’s Republic of China*, listed manufacturing enterprises should publish their conduct of social responsibility in time and make detailed disclosure on their environmental responsibilities. Manufacturing enterprises should evaluate the impact of their behavior on the natural environment, actively undertake their environmental responsibilities and promote sustainable development. Multiple pressures from the public, the capital market, and the government compel enterprises to undertake effective environmental management measures as a response to the demands of environmental supervision [34]. CER increases heavily polluting companies’ long-term economic effects through improving companies’ operational efficiency and reducing their credit costs [18]. It brings sufficient financial support for corporate innovation.

First, we argue that environmental regulations can promote enterprises to take environmental responsibility. Environmental regulation is conducive to guiding listed companies to strengthen environmental management system certification and environmental information disclosure [35]. According to the stakeholder theory, environmental information disclosure wins favor from investors and protection from supervisors, thereby reducing the risks of investment and promote transparency [36]. Inspired by stakeholders, enterprises prefer to pay attention to CER, carry out active measures of environmental governance, and increase investment in environmental protection [37]. Environmental supervision has certainly changed corporate behavior, promoting business executives to carry out different environmental strategies in response to various types of environmental regulation [38,39].

Second, we argue that CER can promote green innovation. Green innovation demands more regulatory commitment and concern than other types of innovation. Environmental responsibility can encourage companies to allocate more resources to green innovation when making business decisions [22]. Several past studies suggest that green strategy and green human resource management have a positive influence on the green process and product innovation [17,40]. Tseng et al. [41] suggest that environmental responsibility is the foundation of sustainable development, which drives corporate financial and governance performance. Mishra [42] shows that innovative enterprises benefit from engaging in CSR activities through their environmental dimensions. Environmental responsibility can promote the enterprise to form the concept of green development and encourage managers and staff’s identification with environment protection awareness. Transformation of corporate R&D mode requires the corresponding transformation of a green organization, i.e. the company’s inner procedure should be redesigned with additional development of green innovation capability [27]. Enterprises’ internal features define the effects of environmental regulation policies on environmental performance. Environment management system certification is beneficial to the formation of a corporate green innovation culture and encourages activities of green technology innovation. In addition, corporate environmental responsibility also improves the reputation of society and enables it to receive more external support. Based on the above, this paper proposes the following hypotheses:

Hypothesis 2: Environmental regulation impels manufacturing enterprises to improve environmental responsibility.
Hypothesis 3: Corporate environmental responsibility strengthens the relationship between environmental regulation and green technology innovation.

**Fig 1:**
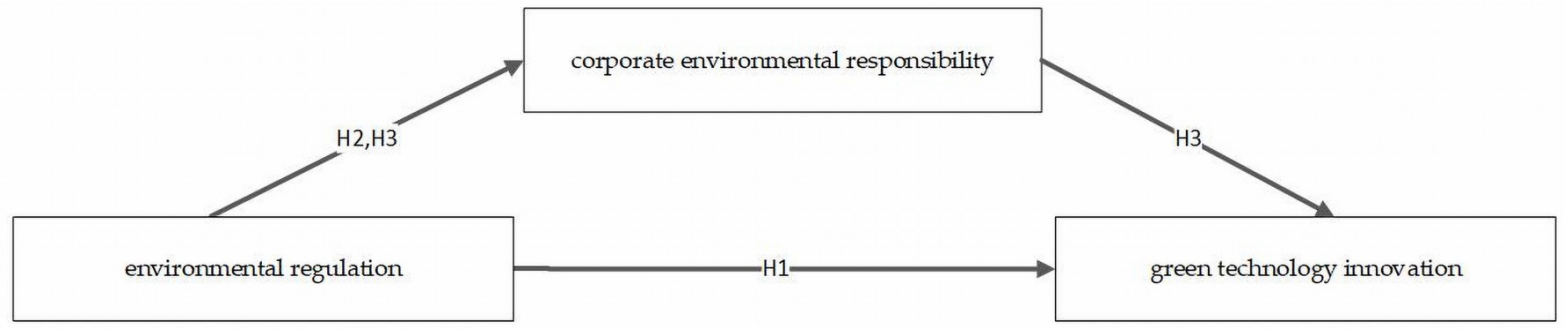
Research framework.

This paper tested the proposed relationships as per the framework shown in Fig 1.

## Data and methodology

### Data source and descriptive statistics

According to the *Guideline for Environment Information Disclosure of Listed Companies* released by China’s Ministry of Environmental Protection, the heavily polluting industries include 16 industries in total: thermal power generation, steel making, cement, electrolytic aluminum, coal mining, metallurgy, chemical engineering, petrochemical engineering, construction materials, paper-making, brewing, pharmaceutical production, fermentation, textiles, tannery, and mining. From the State Intellectual Property Office of China (SIPO), this paper retrieves the patent application and patent authorization of listed enterprises in the heavy pollution industry. Green patent classification numbers are then determined according to the list of green patent classifications issued by the World Intellectual Property Organization (WIPO). We collected the heavily polluting manufacturing listed firms (excluding ST and *ST firms) from the China Stock Market Accounting Research (CSMAR) database. The data contains financial data and characteristics of firms listed in the Shanghai and Shenzhen Stock Exchange. CER score and CSR score of listed companies come from the Hexun website. The sample excludes firms with missing core variables. Since most companies began to disclose environmental information in 2010, we set the starting year of the data to 2010. We have manually sought data of pollution charges from annual environment reports and financial statements of listed companies. Our final sample consists of 3121 firm-year observations during the period 2010 to 2017.

### Model construction

Based on the Mediating Effect Model of Baron and Kenny [43] and the research design of Li and Xiao [38], here we construct the following metrological model:

$${\text{GTI}}_{\text{i},\text{t}}=\alpha_{1}+\theta_{1}{\text{Charge}}_{\text{i},\text{t}}+\sum \gamma_{\text{k}}{\text{Control}}_{\text{k},\text{i},\text{t}}+\delta_{0}{\text{Industry}}_{\text{i}}+\delta_{1}{\text{Year}}_{\text{t}}+\delta_{2}\text{Region}+\varepsilon_{\text{i},\text{t}}$$ (1)

$${\text{CER}}_{\text{i},\text{t}}=\alpha_{2}+\theta_{2}{\text{Charge}}_{\text{i},\text{t}}+\sum \gamma_{\text{k}}{\text{Control}}_{\text{k},\text{i},\text{t}}+\delta_{0}{\text{Industry}}_{\text{i}}+\delta_{1}{\text{Year}}_{\text{t}}+\delta_{2}\text{Region}+\varepsilon_{\text{i},\text{t}}$$ (2)

$${\text{GTI}}_{\text{i},\text{t}}=\alpha_{3}+\theta_{3}{\text{Charge}}_{\text{i},\text{t}}+\theta_{4}{\text{CER}}_{\text{it}}+\sum \gamma_{\text{k}}{\text{Control}}_{\text{k},\text{i},\text{t}}+\delta_{0}{\text{Industry}}_{\text{i}}+\delta_{1}{\text{Year}}_{\text{t}}+\delta_{2}\text{Region}+\varepsilon_{\text{i},\text{t}}$$ (3)

$${\text{GTI}}_{\text{i},\text{t}}=\alpha_{4}+\beta_{0}{\text{Charge}}_{\text{i},\text{t}}+\beta_{1}{\text{RD}}_{\text{i},\text{t}}+\beta_{2}{\left(\text{Charge}*\text{RD}\right)}_{\text{i},\text{t}}+\sum \gamma_{\text{k}}{\text{Control}}_{\text{k},\text{i},\text{t}}+\delta_{0}{\text{Industry}}_{\text{i}}+\delta_{1}{\text{Year}}_{\text{t}}+\delta_{2}\text{Region}+\varepsilon_{\text{i},\text{t}}$$ (4)

$${\text{GTI}}_{\text{i},\text{t}}=\alpha_{5}+\tau_{0}{\text{Charge}}_{\text{i},\text{t}}+\tau_{1}{\text{ENVI}}_{\text{i},\text{t}}+\tau_{2}{\left(\text{Charge}*\text{ENVI}\right)}_{\text{i},\text{t}}+\sum \gamma_{\text{k}}{\text{Control}}_{\text{k},\text{i},\text{t}}+\delta_{0}{\text{Industry}}_{\text{i}}+\delta_{1}{\text{Year}}_{\text{t}}+\delta_{2}\text{Region}+\varepsilon_{\text{i},\text{t}}$$ (5)

Benchmark model (1) is constructed based on Hypothesis 1, with subscript indexes *i* and t representing the enterprise and the year respectively. GTI serves as the explanatory variable, representing the green technology innovation level of listed manufacturing enterprises. The Charge is the explanatory variable representing the environmental regulation for enterprises. Model (2) and model (3) are constructed based on Hypotheses 2 and 3. CER is a mediating variable representing the level of corporate environmental responsibility, α is the constant term, and ε is the random disturbance term. The Control_k, i, t_ is a series of controlled variables, including the share of the largest shareholder (SHR1), asset size (SIZE), asset-liability ratio (LEV), the proportion of male executives (GENDER), CEO duality (DUAL), state ownership (STATE), return on assets (ROA), and operating life (AGE). In addition, we control the annual (Year), industry (Industry), and regional (Region) fixed effect.

It should be mentioned that *θ*_1_ is the total effect of environmental regulation on corporate green innovation; *θ*_2_ is the impact of environmental regulation on corporate environmental responsibility; *θ*_3_ is the direct effect of environmental regulation on corporate green innovation; *θ*_4_ is the impact of environmental responsibility on corporate green innovation after environmental regulation is under control. *θ*_2_*θ*_4_ represents the mediating effect transmitted via corporate environmental responsibility, i.e. the indirect effect.

For further research, model (4) and model (5) are constructed to discuss the moderating effect of R&D expenditure and environment protection investment under environmental supervision. We use the ratio of R&D expenditure to main business income to evaluate RD and use the natural logarithm of the enterprise environmental investment to measure ENVI.

### Explanation of variables

#### Explained variable

We use the natural logarithm of patents authorization plus one to measure the green technology innovation (GTI), including the green invention and green utility patents. Although data on patent applications are more timely, the number of green patents authorized can better reflect the true innovation level of an enterprise [44,45].

#### Explanatory variable

Environmental regulation is mainly measured by indicators, such as investment expenditure in environmental governance, composite emission index, or numbers of environmental violations [2,3,7,38]. Most measurements are based on macro-data at the city level or above, which could not be directly used to measure the environmental supervision of the company [46]. Following Li and Xiao [38], this study uses the proportion of pollution charge (Charge) to total assets of listed manufacturing enterprises to measure the degree of environmental regulation. The system of pollution charge has been widely applied in China, motivating enterprises to incorporate environmental responsibility into production decisions. As an environmental regulation measure targeting enterprises, the governance effect created by billing pollution discharge is immediately evident.

#### Mediation variables

We take CER as the mediating variable, measured by the ratio of CER to CSR of listed manufacturing enterprises. The CSR score of listed companies includes the dimensions of shareholder responsibility, employee responsibility, supplier, customer and consumer rights and interests, environmental responsibility, and social responsibility. CER score is calculated based on assessments of listed companies’ environment governance actions, including environment protection awareness, environmental management system certification, environment protection investment, and energy conservation. These dimensions comprehensively reflect a listed company’s level of environment governance [47].

#### Other control variables

Enterprise innovation is affected by factors such as company characteristics, board characteristics, and so on. We add these variables to control for unobserved heterogeneity as follows.

Share of the largest shareholder (SHR1): The ownership concentration ratio is measured by the shareholding ratio of the first majority shareholder. Based on the Principal-Agent Theory, senior management is more likely to be affected by the interests of major shareholders.
Asset size (SIZE): The size of Enterprises is measured by natural logarithms of corporate total assets. The large company attracts more government and public attention [1].
Asset-liability ratio (LEV): The asset-liability ratio is a comprehensive index to evaluate the company’s debt and risk level. Low financial risk is conducive to enterprises to increase R&D expenditure and environmental protection investment.
The proportion of male executives (GENDER): Under external pressure, senior executives of different genders place different levels of emphasis on corporate innovation and environmental performance [48].
CEO duality (DUAL): 1 if the CEO also chairs the board and 0 otherwise. CEO duality improves the company’s operational efficiency, a powerful CEO will result in greater innovation performance [49].
State ownership (SOE): 1 if enterprise for the state-owned and 0 otherwise. Enterprises with different ownership systems have different governance characteristics, which affect the policy effects of environmental regulation.
Return On Assets (ROA): Return on assets often represents the company’s performance and own profitability.
Operating life (AGE): The life cycle of a company will not only affect the business performance but also indirectly affect innovation. We use the age of the company to measure its operating life.

## Main results

### Descriptive statistics

Table 1 is a statistical description of the main variables in this paper. More than half of the heavily polluting enterprises have no green patents, and their innovation outputs are mainly non-green ones. Enterprises with more green patent authorization are chemical raw materials, chemical products manufacturing, and pharmaceuticals, ferrous metal smelting, and rolling procession, nonferrous metal mining, and dressing. Industries with higher marks of environmental responsibility include chemical products manufacturing, pharmaceuticals, and beverage manufacturing. Industries with higher profit margins are pharmaceuticals and chemical products manufacturing. The average pollution charge is 0.036(%), indicating that listed heavy-pollution manufacturing enterprises spend a large amount of money on pollution discharge. And the enterprises that pay the pollutant discharge fees account for a relatively low proportion. We find that manufacturing enterprises with excellent performance of green innovation, environmental responsibility, and financial results are mostly of the chemical products manufacturing, pharmaceuticals, and beverage manufacturing.

**Table 1:** Descriptive statistics.

Variable	Definition	Observations	Mean	Median	Standard deviation
GTI	Green technology innovation	3121	0.148	0	0.426
Charge	Pollution charges	3121	0.036	0	0.087
CER	Environmental responsibility	3121	0.043	0	0.102
SHR1	Share of the largest shareholder	3121	0.36	0.345	0.144
SIZE	Asset size	3121	21.756	21.647	1.135
LEV	Asset–liability ratio	3121	0.387	0.372	0.204
GENDER	Proportion of male executives	3121	0.836	0.85	0.105
DUAL	CEO duality	3121	0.251	0	0.433
SOE	Ownership attribute	3121	0.402	0	0.49
ROA	Return On Assets	3121	0.054	0.045	0.05
AGE	Operating life	3121	15.729	15.77	5.105

Based on whether companies pay pollution charges, we conduct a T-mean difference test of sample groups (Table 2). For enterprises not paying pollution charges (G1), their GTI average and CER average are half of those that pay pollution charges (G2). Their difference in mean value and median is at the significant level of 1%. Remarkably, enterprises paying pollution charges are better at green innovation and environmental responsibility performance.

**Table 2:** T-mean difference test of sample groups.

Variables	G1	Mean1	G2	Mean2	MeanDiff
GTI	2112	0.113	1009	0.222	-0.108***
CER	2112	0.033	1009	0.064	-0.030***

Note:

***Significance level at the 1%.

### Analysis of the main model’s regression results

Columns (1)—(3) in Table 3 are the regression results of models (1) to (3) respectively. The regression coefficient of environmental regulation on heavily polluting manufacturing enterprises’ green technology innovation is 0.297, at the significant level of 1%. In other words, should the percentage of pollution charges incorporate total assets increase by 1%, the number of green patents held by these enterprises will increase by 0.297%. Overall, the environmental regulation has a promoting effect on the green innovation activities of Chinese heavily polluting manufacturing enterprises, consistent with Hypothesis 1. In reality, innovation is the sole solution for enterprises to gain competitive gains and reduce costs of environmental regulation.

**Table 3:** Regression analysis of the main model.

Variable	(1)	(2)	(3)
	GTI	CER	GTI
Charge	0.297***	0.0503**	0.284***
	(0.109)	(0.0219)	(0.108)
CER			0.299***
			(0.102)
SHR1	-0.125**	-0.0005	-0.125**
	(0.0502)	(0.0120)	(0.0497)
SIZE	0.0791***	0.0215***	0.0726***
	(0.0099)	(0.0019)	(0.0097)
LEV	0.0839*	-0.0093	0.0867*
	(0.0465)	(0.0107)	(0.0464)
GENDER	0.320***	0.0401***	0.308***
	(0.0596)	(0.0150)	(0.0593)
DUAL	-0.0078	0.0011	-0.0081
	(0.0156)	(0.0035)	(0.0156)
SOE	0.0152	0.0247***	0.0078
	(0.0177)	(0.0040)	(0.0176)
ROA	-0.233	0.0133	-0.237
	(0.158)	(0.0368)	(0.157)
AGE	-0.0081***	-0.0009**	-0.0078***
	(0.0016)	(0.0004)	(0.002)
Constant	-1.708***	-0.388***	-1.592***
	(0.205)	(0.042)	(0.199)
Year	Yes	Yes	Yes
Industry/Region	Yes	Yes	Yes
Observations	3,121	3,121	3,121
R-squared	0.129	0.231	0.133

Note:

***, **, and * denote statistical significance at the 1%, 5%, and 10% levels, respectively.

Columns (2) and (3) in Table 3 show the regression results of the mediating effect. The regression coefficient of environmental regulation on CER is 0.05, at the significant level of 5%. In other words, should the percentage of pollution charges incorporate total assets increase by 1%, the score of environmental responsibility by these enterprises will increase by 0.05%. Environmental regulation can strengthen heavily polluting enterprises’ environmental responsibility, consistent with Hypothesis 2. The regression results demonstrate that the impacts of both environmental regulation and environmental responsibility on green technology innovation are remarkably positive.

It demonstrates the existence of the “partial mediating effect” of corporate environmental responsibility, namely, part of environmental regulation’s stimulating effect on green technology innovation is achieved by strengthening CER. This paper proves that, as an important channel to affect corporate management in the form of environmental regulation, environmental responsibility can effectively improve a company’s level of green innovation. CER includes environmental protection awareness, environmental management system certification, environmental investment, and other aspects, and the impact of these factors on green technology innovation is positive. The results demonstrate that CER has strengthened the relationship between environmental regulation and green technology innovation. Performing environmental responsibility and developing green innovation are the main measures of enterprises in solving environmental problems.

We use the stepwise regression method to verify the mediating effect and continues to use the Sobel test to examine the mediating effect of CER. The Sobel test results are shown in Table 4. The Z statistic of indirect effects is significant at the 5% level, indicating that there is a mediating effect, which accounts for 5.4%. At the same time, the results of the Sobel test are consistent with the results of the full-sample regression.

**Table 4:** The Sobel test result.

	Coefficient	Standard deviation	Z	P>Z
Indirect effect	0.017	0.007	1.974	0.048
Direct effect	0.300	0.085	3.567	0.000
Total effect	0.317	0.085	3.726	0.000
The ratio of mediating effect = 5.4%				

The results of the control variables are summarized as follows. The share of the largest shareholder (SHR1) and operating life (AGE) both have a significant negative effect on GTI, suggesting that higher ownership concentration and operating life go against green innovation. The asset size (SIZE) and asset-liability ratio (LEV) both have a significant positive effect on GTI, suggesting that companies with large scale and better financial situations pay more attention to green innovation. The gender ratio (GENDER) has a significant positive effect on innovation, which means that male executives are more concerned with green innovation and environmental responsibility. The coefficients of CEO duality (DUAL) and accounting earnings (ROA) are not significant. Besides, there is no clear distinction between state-owned enterprises’ level of green technology innovation and that of private enterprises.

### Robustness test and instrumental variable test

In this paper, we conduct robustness tests as follows: (1) replacing independent variable and dependant variable respectively; (2) lag one-year processing on independent variables and control variables; (3) adopting Logit regression for empirical analysis. First, we select the ratio of corporate charges to regional sewage charges as a substitute indicator for environmental regulation. Then, we change the dependant variable, representing the corporate level of green technology innovation with the number of authorized patents on green utility models. Considering the lagging impact of environmental policy, this paper conducts lag one-year processing on independent variables and control variables to retest the model (1). In addition, we define corporate green technology innovation as a dummy variable T and use Logit regression for empirical analysis. The results of our robustness tests are given in Table 5. We find that pollution charge (Charge) is still significant and positive for green technology innovation in the model (1).

**Table 5:** Regression results of robustness tests based on another measurement.

Variable	(1)	(2)	(3)	(4)
	Replacing Charge	Replacing GTI	Lag one year	Logit model
	GTI	GTI	GTI	GTI
Charge	0.980**	0.207**	0.266*	1.937***
	(0.474)	(0.0917)	(0.140)	(0.653)
SHR1	-0.112**	-0.102***	-0.198***	-1.098***
	(0.0503)	(0.0352)	(0.0700)	(0.424)
SIZE	0.0695***	0.0308***	0.0738***	0.479***
	(0.0098)	(0.0073)	(0.0129)	(0.0721)
LEV	0.0965**	0.0770**	0.148**	0.731
	(0.0467)	(0.0375)	(0.0712)	(0.445)
GENDER	0.307***	0.258***	0.402***	3.426***
	(0.0593)	(0.0485)	(0.0833)	(0.739)
DUAL	-0.0079	0.0092	-0.0180	-0.159
	(0.0155)	(0.0142)	(0.0227)	(0.163)
SOE	0.0162	0.0130	0.0404	0.105
	(0.0177)	(0.0138)	(0.0255)	(0.148)
ROA	-0.209	0.0445	-0.260	-2.458
	(0.155)	(0.139)	(0.231)	(1.508)
AGE	-0.0073***	-0.0042***	-0.0069***	-0.0493***
	(0.00156)	(0.0013)	(0.0022)	(0.0145)
Constant	-1.560***	-0.725***	-1.654***	-16.77***
	(0.206)	(0.150)	(0.278)	(1.742)
Year	YES	YES	YES	YES
Industry/Region	YES	YES	YES	YES
Observations	3,121	3,121	1,799	2,604
R-squared	0.135	0.074	0.155	

Note:

***, **, and * denote statistical significance at the 1%, 5%, and 10% levels, respectively.

The results listed in Table 5 show that the regression coefficient of pollution charge on GTI is remarkably positive and consistent with previous conclusions. It also indicates that enterprises should pay more attention to green technology innovation and environmental responsibility in the future in response to environmental regulation.

To deal with the possible endogeneity between environmental regulation and GTI, Our manuscript reports the results of a robustness test based on the two-stage instrumental variable method and GMM instrumental variable method (Table 6). We choose the word frequency of environmental information disclosure in company quarterly and annual reports and the average annual pollution charge of other listed companies in the industry as the instrumental variables for Charge. Table 6 shows that the pollution charge of heavily polluting listed companies in China significantly increases their green technology innovation.

**Table 6:** Results based on two-stage and GMM instrumental variable method.

VARIABLES	(1)	(2)
	2SLS	GMM
	GTI	GTI
Charge	2.415***	2.577***
	(0.452)	(0.457)
SHR1	-0.178***	-0.146***
	(0.0544)	(0.0545)
SIZE	0.0871***	0.0832***
	(0.0106)	(0.0107)
LEV	-0.0363	-0.0668
	(0.0523)	(0.0527)
GENDER	0.260***	0.259***
	(0.0693)	(0.0706)
DUAL	-0.0198	-0.0179
	(0.0186)	(0.0189)
SOE	0.00556	0.0152
	(0.0199)	(0.0201)
ROA	-0.110	-0.209
	(0.175)	(0.175)
AGE	-0.0088***	-0.0089***
	(0.00171)	(0.00173)
Constant	-1.779***	-1.763***
	(0.216)	(0.219)
Year	YES	YES
Industry/Region	YES	YES
Observations	3,120	3,120

Note:

***, **, and * denote statistical significance at the 1%, 5%, and 10% levels, respectively.

## Further discussion

### R&D expenditure and environment protection investment

The mediating effect test shows that the indirect effect of environmental regulation on innovation is lower than the direct effect. For further investigation, this study discusses enterprises’ responses to environmental regulation under two situations. First, enterprises can increase their R&D expenditure and launch green innovation or other kinds of innovation for promoting green development and competitiveness. Secondly, enterprises can also increase their environment protection investment for either environmental responsibility or green innovation, to reduce environmental pollution and improve environmental performance. According to theoretical analysis, enterprises can choose both green technology innovation and non-green technology innovation to enhance their competitiveness under the pressure of environmental regulation. The results of our empirical analyses concerning interaction terms are given in Table 7.

**Table 7:** The moderating role of R&D expenditure and environment protection investment.

Variable	(1)	(2)
	GTI	GTI
Charge	0.0969	0.258**
	(0.111)	(0.110)
Charge×RD	6.627*	
	(3.784)	
RD	1.442***	
	(0.348)	
Charge×ENVI		0.0162
		(0.0419)
ENVI		0.0117**
		(0.00511)
SHR1	-0.132***	-0.127**
	(0.0482)	(0.0499)
SIZE	0.0756***	0.0759***
	(0.0095)	(0.0099)
LEV	0.0609	0.0834*
	(0.0459)	(0.0464)
GENDER	0.227***	0.309***
	(0.0620)	(0.0597)
DUAL	-0.0121	-0.0075
	(0.0154)	(0.0155)
SOE	0.0327*	0.0116
	(0.0175)	(0.0177)
ROA	0.0116	-0.242
	(0.158)	(0.157)
AGE	-0.0056***	-0.0077***
	(0.0016)	(0.0016)
Constant	-1.672***	-1.629***
	(0.204)	(0.205)
Year	YES	YES
Industry/Region	YES	YES
Observations	3,121	3,121
R-squared	0.156	0.134

Note:

***, **, and * denote statistical significance at the 1%, 5%, and 10% levels, respectively.

The interactive Charge × RD is significantly positive, the interactive Charge × ENVI is positive but not significant. This result shows that corporate R&D expenditure has strengthened the stimulus given by pollution charge on corporate green innovation, and thus performing a positive moderating role. Manufacturing enterprises can strengthen green technology innovation by increasing R&D expenditure and environment protection investment.

### Heterogeneity analysis

Political connection is more than just an important route affecting corporate innovation. It also affects the execution of environmental regulations. On the one hand, private enterprises and weak technology companies have enhanced their innovation capabilities via political connections [50]. CEO’s support for environmental protection policies also helps promote corporate green technology innovation [51]. The stricter the environmental regulation, the higher the deployment effect of politically-related R&D resources, which in turn improves R&D effects [52]. On the other hand, political connections tend to reduce the due punishments of environmental violations and lead to the “protection effect”. Strong political connections amplify enterprises’ advantages at bargaining, and thus restrains environmental regulation from uplifting the total green factor productivity dynamically [15]. Political connections of CEOs could also strengthen the execution of environmental regulation policies, amplifying the policy effect of environmental regulation. In this paper, we categorize samples into those politically connected and those not, based on whether their CEO is a Communist Party member, Deputy to the National People’s Congress, or member of the National Committee of the Chinese People’s Political Consultative Conference. Column (1)–(2) in Table 8 reports that the pollution charge has a greater positive role in the green innovation of enterprises that lack political connection. The political associations of CEO somewhat suppress the policy effect of environmental regulation.

**Table 8:** Heterogeneity test of political connection and environmental information disclosure.

Variable	(1)	(2)	(3)	(4)	(5)	(6)
	Political connection		Environmental report		Information disclosure	
	No	Yes	No	Yes	No	Yes
	GTI	GTI	GTI	GTI	GTI	GTI
Charge	0.541***	0.197*	0.210*	0.440***	0.214**	0.443**
	(0.209)	(0.121)	(0.138)	(0.170)	(0.108)	(0.197)
SHR1	-0.0025	-0.147**	-0.160**	-0.0924	-0.132**	-0.136
	(0.0755)	(0.0747)	(0.0664)	(0.0879)	(0.0550)	(0.108)
SIZE	0.0315**	0.100***	0.0836***	0.0666***	0.0765***	0.0807***
	(0.0132)	(0.0143)	(0.0149)	(0.0145)	(0.0121)	(0.0189)
LEV	0.0785	0.123*	-0.0530	0.193**	0.0773	0.0829
	(0.0706)	(0.0721)	(0.0704)	(0.0793)	(0.0574)	(0.0857)
GENDER	0.355***	0.265***	0.282***	0.260***	0.332***	0.307**
	(0.0865)	(0.0908)	(0.0900)	(0.0971)	(0.0635)	(0.132)
DUAL	-0.0375*	-0.0024	0.0117	-0.0299	0.0032	-0.0398
	(0.0202)	(0.0281)	(0.0244)	(0.0241)	(0.0178)	(0.0308)
SOE	0.0935**	-0.0181	0.0021	0.0462	0.0097	0.0268
	(0.0397)	(0.0261)	(0.0263)	(0.0282)	(0.0213)	(0.0331)
ROA	0.289	-0.585**	-0.297	0.0962	-0.406**	-0.00801
	(0.271)	(0.236)	(0.207)	(0.289)	(0.166)	(0.344)
AGE	-0.00144	-0.0119***	-0.0052**	-0.0086***	-0.0068***	-0.0112***
	(0.0019)	(0.0025)	(0.0026)	(0.0023)	(0.0018)	(0.0030)
Constant	-0.991***	-2.054***	-1.811***	-1.596***	-1.556***	-1.913***
	(0.271)	(0.307)	(0.324)	(0.318)	(0.253)	(0.393)
Year	YES	YES	YES	YES	YES	YES
Industry/Region	YES	YES	YES	YES	YES	YES
Observations	1,043	1,713	1,260	1,496	2,095	1,026
R-squared	0.148	0.170	0.165	0.142	0.125	0.179

Note:

***, **, and * denote statistical significance at the 1%, 5%, and 10% levels, respectively.

External pressure is an important reason for pollution charges to drive the green innovation of enterprises [38]. Such a disclosure mends the issue of unbalanced information between enterprises and investors, reducing the financing restraints and costs for a company [53]. The excellent financial condition helps enterprises carry out their duties of environmental governance and implement green innovation. Accordingly, the more transparent the environment disclosure, the more stimulation of environmental regulation on corporate innovation [54]. Here we categories sampled enterprises into two groups based on whether they publish annual environmental reports and conduct examination. This paper conducts a group regression of sample companies according to whether the number of environmental information disclosure items exceeds the industry average. The results of Columns (3) to (6) in Table 8 show that the pollution charge is positively correlated with GTI, significantly at the 5% level. In the group of disclosing public environmental annual reports, the regression coefficient of pollution charges is 0.44, while the regression coefficient of companies that does not disclose environmental reports is 0.21. Pollution charge has a greater incentive for green innovation in manufacturing companies that disclose environmental information promptly. Green innovation and environmental responsibility are mutually beneficial, manufacturing enterprises with better environmental responsibility tend to achieve better policy effects of environmental regulations. As is part of environmental responsibility, environmental information disclosure has also strengthened the relationship between environmental regulations and corporate innovation.

In China, the central government’s policies of environmental regulation serve as the foundation for regional governments to draft their respective policies. Considering regional differences, the central government has classified them into various functional zones, each bearing a unique task of ecological protection and with the respective goal of economic development. Some regional governments aim at maximized economic benefits and often loosen the restraints of environment protection for economic advancement, other regional governments aim at ecological assessment, hence stricter execution of environmental regulations. Even within the same province, cities differ in their execution of environmental regulation. According to Du et al. [55], the transformation of regional governance contributes to the realization of the “Porter Hypothesis” in the Chinese context and increases industrial green competitiveness. The size of a government is closely related to environmental regulation. A minor government and loose regulation have reduced enterprises’ environmental responsibilities [33]. Regional features like governance goals and financial decentralization affect the effect of implementing environmental regulation policies. Environmental regulation is under the sway of external circumstances, and competition among regional governments will alter the degree to which they implement environmental regulations. For instance, neighboring districts are economically competing against each other, yet they are also imitating each other in terms of drafting and implementing environmental regulation policies [56]. Therefore, the external governance environment will change the degree of impact of environmental regulations on corporate green innovation.

We continue to discuss the impact of external governance factors on the implementation of environmental regulations like regional government’s environment restraint goals and financial decentralization. Following Yu et al. [57], we collect data of environment goal restraint disclosed in every city governments’ reports. According to whether the local government of the enterprise puts forward the target of industrial pollution reduction, the sample data has been divided into two groups. At the same time, we also classify the sample into two groups according to whether the fiscal decentralization of the company’s location exceeds the median of the year. Table 9 is the regression results of how pollution charge affects corporate GTI under different regional governance characteristics. We find that the regression coefficient of pollution charge on GTI of heavily polluting firms is still significantly positive. In low-environment target cities, environmental regulations have a greater impact on the green innovation of manufacturing companies in heavily polluting industries.

**Table 9:** Heterogeneity test of regional differences.

Variable	(1)	(2)	(3)	(4)	(5)	(6)
	Environmental objectives		Fiscal decentralization		Eastern	Central, West
	Low	High	Weak	Strong		
	GTI	GTI	GTI	GTI	GTI	GTI
Charge	0.394***	0.236*	0.422***	0.178*	0.337**	0.136
	(0.144)	(0.161)	(0.161)	(0.143)	(0.132)	(0.196)
SHR1	-0.0508	-0.215***	-0.244***	-0.0745	0.0145	-0.266***
	(0.0744)	(0.0679)	(0.0800)	(0.0664)	(0.0655)	(0.0717)
SIZE	0.0782***	0.0807***	0.0618***	0.0966***	0.0588***	0.102***
	(0.0135)	(0.0147)	(0.0154)	(0.0133)	(0.0120)	(0.0160)
LEV	0.107	0.0120	0.169***	-0.00261	0.0679	0.103
	(0.0706)	(0.0638)	(0.0627)	(0.0686)	(0.0573)	(0.0751)
GENDER	0.352***	0.198**	0.439***	0.156*	0.372***	0.168
	(0.0906)	(0.0877)	(0.0934)	(0.0882)	(0.0701)	(0.103)
DUAL	-0.0167	-0.0116	0.0352	-0.0500**	0.0093	-0.0642***
	(0.0250)	(0.0195)	(0.0223)	(0.0234)	(0.0194)	(0.0246)
SOE	0.0177	0.0130	-0.0712***	0.0817***	-0.0474**	0.100***
	(0.0258)	(0.0255)	(0.0269)	(0.0239)	(0.0227)	(0.0280)
ROA	-0.444**	0.186	-0.0233	-0.522**	-0.208	-0.170
	(0.220)	(0.235)	(0.204)	(0.243)	(0.179)	(0.307)
AGE	-0.0094***	-0.0037*	-0.0057***	-0.0111***	-0.0036*	-0.0141***
	(0.0024)	(0.002)	(0.0020)	(0.0024)	(0.0018)	(0.0028)
Constant	-1.841***	-1.668***	-1.406***	-1.996***	-1.384***	-2.028***
	(0.284)	(0.310)	(0.311)	(0.278)	(0.252)	(0.319)
Year	YES	YES	YES	YES	YES	YES
Industry/Region	YES	YES	YES	YES	YES	YES
Observations	1,467	1,654	1,536	1,585	1,852	1,269
R-squared	0.176	0.122	0.126	0.180	0.091	0.211

Note:

***, **, and * denote statistical significance at the 1%, 5%, and 10% levels, respectively.

In regions with strong fiscal decentralization, environmental regulations have a weaker impact on green innovation in manufacturing companies in heavily polluting industries. Environmental regulation policy depends on the execution of respective governments, and regional governments with a higher degree of financial decentralization tend to be more capable at execution.

In China, the eastern, central, and western regions have differences in the market environment and economic development, thus affecting the implementation effect of environmental policies. In the results of Column (5) and Column (6), we find environmental regulation has a greater impact on GTI in eastern China. The heterogeneity test of regional differences suggests that, as a market-incentive environmental regulation policy, the pollution charge is less dependent on the government’s executive power.

## Conclusions

According to the Porter Hypothesis, moderate environmental regulation can make the internalization of external issues like corporate pollution and encourage corporate innovation activities. In particular, the effect of environmental policy on innovation depends on the policy means and the strategic behavior of the enterprise [58,59]. This paper analyses the transmission mechanism of how environmental regulation affects corporate environmental responsibility and green technology innovation, in addition to the theoretical discussion on how various governance conditions adjust environmental regulation. Pollution charge is a market-incentive environmental regulation method that directly faces enterprises, which produces higher regulatory effects. Compelled to pay, heavily polluting manufacturing enterprises have to strengthen their environmental responsibility and green technology innovation. In the meantime, increasing R&D expenditure and environment protection investment is better to enhance the effect of implementing environmental regulation policies, thus promoting the green innovation output of manufacturing enterprises. The research in this article shows that corporate environmental responsibility is closely related to green innovation, which theoretically guides the sustainable development of manufacturing companies.

The study makes a profound analysis of the relation of pollution charges on green technology innovation by using microdata of heavily polluting manufacturing enterprises listed in Chinese A Stock Markets, providing a more targeted reference for making policies. We have manually collected information on corporate pollution charges, corporate environmental responsibility, environment protection investment, and green patents, and empirically prove that the mechanism of pollution charges affecting corporate environmental responsibility and green technology innovation. We find that corporate environmental responsibility also significantly enhances its green technology innovation capability via improving manufacturing enterprises’ environmental responsibility. As the main response of manufacturing enterprises to environmental laws and regulations, corporate environmental responsibility has improved the level of green technological innovation of manufacturing enterprises. Further investigation reveals that investment in R&D and environment protection can greatly strengthen the positive effect of environmental regulation on the manufacturing enterprises’ green technology innovation. On the contrary, the CEO’s political connections inhibit the policy impact of pollution charges on corporate green technology innovation.

Innovation and environmental responsibility are the directions to promote the circular economy at the corporate level [60,61]. For enterprises that disclose environmental information promptly, environmental regulations have a greater incentive for green technological innovation. The discussion of the heterogeneity of external governance factors shows that in regions with lower environmental goals, environmental regulations have a stronger influence on the green technology innovation of heavily polluting manufacturing enterprises. Environmental regulations have less impact on the green innovation of manufacturing companies in regions with a higher degree of fiscal decentralization. If local governments set higher environmental protection targets, it will be difficult for companies to charge pollutants to stimulate green technological innovation in heavily polluting companies. This paper has examined the mechanism of environmental responsibility’s stimulation on green technology innovation. Such governance behavior includes R&D expenditure, environment protection investment, and environment information disclosure. We have provided new proofs for existing studies on the economic effects of environmental regulation, thus offering theoretical support for the realization of corporate environmental responsibility and green technology innovation. The policy significance of the research conclusions of this paper mainly lies in the following.

First of all, attention should be paid to the construction of a pollution discharge charging mechanism for enterprises. The public sector should optimize the environmental taxation system so that the pollution charges do not become a burden for corporate production. We propose to strengthen the administration of pollution charges and environment taxes, to fully unleash the moderator capacity of market-stimulating environmental regulation on heavily polluting manufacturing enterprises’ environmental responsibility, and to achieve the “positive” effect of environmental regulations on green technology innovation.

In addition, it is urgent to increase R&D expenditure and environmental protection investment. Specifically, environmental protection investment is the performance and result of environmental responsibility. Manufacturing enterprises should improve internal governance and enhance environmental responsibility that strengthens their green innovation activities. Therefore, manufacturing enterprises need to be encouraged and rewarded for undertaking environmental management system certification, disclosing environmental information, and increasing input of environmental governance.

Finally, policymakers should design different environmental regulation policies following each district’s governance features. Meanwhile, it is very necessary to fully consider the institutional environment and governance characteristics of the enterprise location when implementing environmental policy. The sustainable development of enterprises can be promoted only by setting reasonable environmental protection goals and providing financial incentives for green innovation behavior.

## Supporting information

S1 File (CSV)
